# Validation of attenuation imaging coefficient, shear wave elastography, and dispersion as emerging tools for non-invasive evaluation of liver tissue in children

**DOI:** 10.3389/fped.2023.1020690

**Published:** 2023-04-17

**Authors:** Metin Cetiner, Felix Schiepek, Ilja Finkelberg, Raphael Hirtz, Anja K. Büscher

**Affiliations:** Children's Hospital, Pediatrics II, University of Essen, Essen, Germany

**Keywords:** shear wave elastography, attenuation imaging coefficient, shear wave dispersion, liver tissue, children, non-invasive technique, normal value

## Abstract

**Introduction:**

The number of children with acute and chronic liver disease is rising. Moreover, liver involvement may be limited to subtle changes in organ texture especially in early childhood and some syndromic conditions, such as ciliopathies. Attenuation imaging coefficient (ATI), shear wave elastography (SWE), and dispersion (SWD) are emerging ultrasound technologies providing data about attenuation, elasticity, and viscosity of liver tissue. This additional and qualitative information has been correlated with certain liver pathologies. However, limited data are available for healthy controls and have mainly been raised in adults.

**Methods:**

This prospective monocentric study was conducted at a university hospital with a specialization in pediatric liver disease and transplantation. Between February and July 2021, 129 children aged 0-17.92 years were recruited. Study participants attended outpatient clinics due to minor illnesses excluding liver or cardiac diseases, acute (febrile) infections or other conditions affecting liver tissue and function. ATI, SWE, and SWD measurements were performed on an Aplio i800 (Canon Medical Systems) with an i8CX1 curved transducer by two different investigators with long-standing experience in pediatric ultrasound according to a standardized protocol.

**Results:**

Considering multiple potential covariates, we derived percentile charts for all 3 devices relying on the Lambda-Mu-Sigma (LMS) approach. 112 children were considered for further analysis, excluding those with abnormal liver function and under-/overweight (BMI SDS<-1.96/> 1.96, respectively). Age range was 0-17.92 years (mean 6.89±0.50SD), 58% were male. The mean duration of the ultrasound examination (basic ultrasound plus SWE, SWD, and ATI) was 6.67±0.22 minutes and it was well tolerated in 83% (n=92) of cases. While ATI was related to age, SWD was found to depend on BMI SDS, and SWE on abdominal wall thickness and sex. ATI correlated with neither SWE nor SWD, but SWE was correlated with SWD.

**Conclusions:**

Our study provides norm values and reference charts for ATI, SWE, and SWD considering important covariates including age, sex and, BMI. This may help to implement these promising tools into imaging diagnostics of liver disease and to improve the diagnostic relevance of liver ultrasound. In addition, these noninvasive techniques proved to be time-effective and highly reliable, which make them ideal for application in children.

## Introduction

The number of children with acute and chronic liver disease is rising. The prevalence of non-alcoholic fatty liver disease (NAFLD) in children even approximates the numbers known in adults, with 7.6% in the general population and even >30% in the case of obesity (1). NAFLD not only accounts for liver-related morbidity but is also associated with an increased risk of developing metabolic syndrome with type 2 diabetes, cardiovascular disease, and mortality at adult age (2, 3), representing an enormous economic burden. In contrast to obesity-associated liver disease, which is common in industrialized countries and increases over the lifespan, genetic diseases like ciliopathies are characterized by liver involvement already in early childhood (4, 5). Both disease entities require highly accurate screening tools to detect subtle changes in liver tissue at an early stage to allow early diagnosis and—if possible—start of treatment.

Currently, the evaluation of liver tissue is mainly based on ultrasonography, an established, non-invasive technology that is highly applicable in children due to high image resolution given their body composition. However, the informative value of standard ultrasound examinations is limited to organ structure, size, and perfusion. Detailed analysis and quantitative staging of liver fibrosis and steatosis still require invasive liver biopsy and/or MRI examinations which require anesthesia in small children. New emerging ultrasound technologies such as shear wave elastography (SWE), shear wave dispersion (SWD), and attenuation imaging coefficient (ATI) provide imaging quality even surpassing MRI and CT scans with regard to parenchyma and (micro)perfusion of solid organs.

SWE utilizes the speed of the shear wave, which is the lateral tissue displacement by the power-focused ultrasound pulse. It is the most established among these new quantitative ultrasound technologies and reflects parenchymal elasticity. Clinical application has demonstrated the power of SWE in detecting and grading liver fibrosis not only in adults (6) but also in pediatric populations (7). User-friendly SWE is superior and more operational in difficult clinical circumstances compared to the older transient elastography technique (8–14). Some studies have aimed to establish SWE normal values of liver parenchyma in healthy children (15, 16) with contradicting results and have partially neglected important variables.

SWD displays the frequency dependency of shear wave speed, which is affected by parenchymal viscosity. First data indicate that it might be superior compared to SWE in evaluating liver allograft damage (17) and hepatic lobular inflammation in general (18) and discriminating grades of inflammation (19). Its performance in detecting focal liver lesions is under investigation (20).

ATI calculates the percentage of returned ultrasound energy not absorbed by liver tissue to determine the attenuation by organ tissue. Studies on ATI measurements of liver parenchyma in adults indicate its ability to detect and quantify steatosis and to differentiate between mild, moderate, and severe grades (21–27). Furthermore, ATI correlates with the results of MRI-PDFF (magnetic resonance imaging proton density fat fraction) and histopathology representing the gold standard for fat measurement in liver parenchyma (22, 26, 28, 29) and with grayscale grading (30). In this respect, it also surpasses CAP (controlled attenuation parameter) based on FibroScan® systems (30–32).

These high-end ultrasound techniques, especially if combined, may offer an opportunity to evaluate liver parenchyma quantitatively and qualitatively with the highest possible accuracy in one single examination. However, published data are limited to small study cohorts, mainly addressing adult patients, and/or neglecting important variables, such as BMI and age (15, 16).

The aim of our study was to establish normal values for these promising, non-invasive technologies (SWE, SWD, and ATI) in healthy children considering potential influencing factors to provide of a reliable basis for the advanced evaluation of liver parenchyma in pediatric patients with liver disease.

## Material and methods

### Patient recruitment and data collection

**Figure 1:**
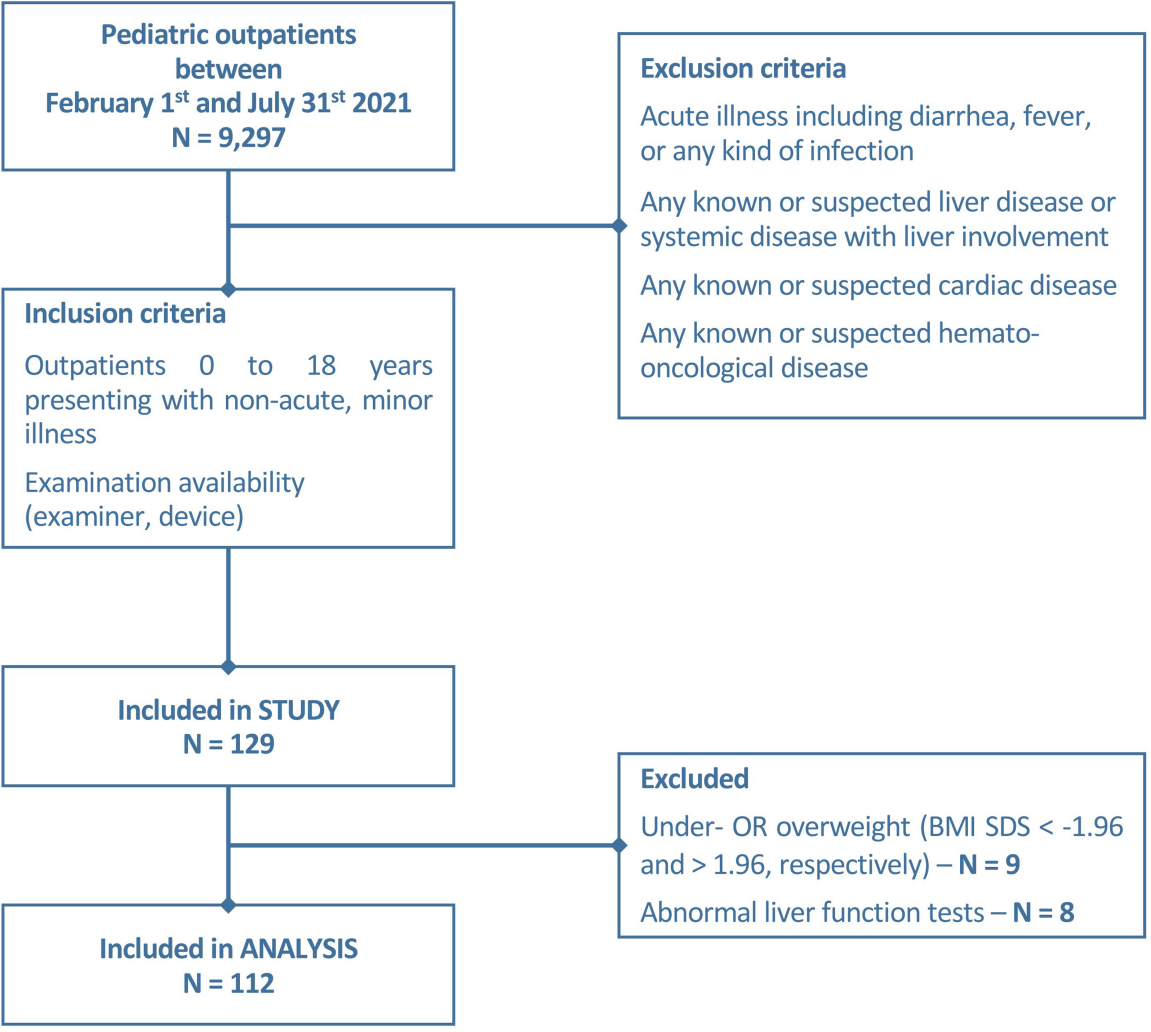
Flowchart describing the recruitment of study patients.

Between February and July 2021, 9,297 patients attended the outpatient clinics of the Children`s Hospital of the University Duisburg-Essen. We consecutively recruited 129/9,297 children (as described in the flowchart, Figure 1) according to the following inclusion criteria: age between 0 and 18 years, examination availability, absence of a medical history or clinical signs of liver, heart, systemic disease, and/or acute illness, including fever. Correspondingly, exclusion criteria were defined as followed: children with any clinical signs of acute illness or with known or suspected liver, cardiac, hemato-oncological diseases or any other systemic diseases with the risk of liver involvement. Non-fasting before the examination was no exclusion criterion. Written informed consent of parents/legal guardians and—if possible—participants was given prior to examination. Clinical (129/129) and laboratory (47/129) data were collected from digital patient records. The study was approved by the local ethics committee (23-11152-BO).

### Standard ultrasound examination

Ultrasound examinations were performed using an Aplio i800 (Canon Medical Systems) with an i8CX1 transducer (PVI-475BT, single curved, 1.8–6.2 MHz). Two pediatricians specialized in pediatric ultrasonography (certified by the German Ultrasound association, DEGUM) and long-standing experience with pediatric liver disease and transplantation performed upon availability and jointly reviewed all examinations.

Examinations were carried out according to a defined protocol: children lay supine with both arms next to the body and were encouraged to breathe calmly (if possible regarding age). The duration of examination, patient cooperation, and last food intake were documented. Standard ultrasound and Doppler examinations included abdominal wall thickness (measured from cutis to peritoneal layer adjacent to liver capsule according to elastography measurement), organ size and shape (liver and spleen), echogenicity and parenchymal texture, including focal or diffuse lesions, dilation of the biliary tract, gallbladder abnormalities, and diameter, velocity and flow profiles of hepatic and splenic arteries and veins. The liver size was measured in the sternal, midclavicular, and anterior axillary line and determined by the mean of all three measurements. The dimension of the spleen was determined below the left costal margin. The results are given as the percentage of age- and height-related normal values (33).

### Shear wave elastography and dispersion of liver tissue

Shear wave speed was measured using an intercostal acoustic window (10 distinct measurements, liver segments V-VIII as recommended (34, 35). Regions of interest (ROI, diameter 1 cm) were placed at 5 cm skin depth (at least 1 cm distance to the liver capsule, avoiding vessels and artifact areas). Mean and standard deviation values are given in kPa and m/s (elastography) and [m/s/kHz] (dispersion).

### Attenuation imaging

Five distinct liver attenuation imaging measurements were performed for every patient (trapezoidal ROI avoiding areas too close to the liver capsule, larger vessels, and artifacts). A quality measure of the liver ATI coefficient that correlates the attenuation with the depth (goodness of fit—R2) was provided. The R2 values were categorized into poor (R2 < 0.80), good (0.80 ≤ R2 < 0.90) and excellent (R2 > 0.90), and only excellent values with R2 > 0.90 were accepted. The mean and standard deviation of the attenuation coefficient in [dB/cm/MHz] are reported.

### Statistical analyses

## Methods

Data handling and statistical analyses were performed with SPSS 25.0 (Armonk, NY: IBM Corp.) and R (version 4.0.3, R Core team, 2020) as well as the R-package FWDselect [version 2.1.0 (36)]. Power analyses were conducted with GPower [3.1, HHU Düsseldorf (37)]. Effect size regarding the employed correlation measures (r) was interpreted according to Cohen et al. (38): small 0.1 ≤ r < 0.3, medium 0.3 ≤ r < 0.5, large r ≥ 0.5.

### Correlation analysis

Prior to analysis, data was semi-winsorized, a procedure by which outliers were replaced by predefined values (39). Following recent recommendations (40), outliers were defined as ATI, SWE (ms and kPa), and SWD measurements exceeding ± 2.5 the median absolute difference (MAD) regarding the respective sonography parameter and replaced by values corresponding to ±2.5 times the respective MAD.

Correlation analyses (Pearson correlation *r*, point-biserial correlation *r_pb_*_,_ Kendall *τ*) were performed considering the scale of measure and the presence of outliers. Linearity between continuous variables was assessed by visual inspection of bivariate scatter plots. The analysis of bivariate correlations was deemed significant at *p* < .05 and exploratory.

### Covariate subset selection

Considering the large number of potential covariates (age, sex, BMI SDS, height-SDS, abdominal wall thickness, liver size (%), spleen size (%), fasting duration, cooperation as well as SWD depth in case of construction of SWD charts) we used a two-step procedure as implemented in the FWRselect R-package to identify the most appropriate subset of covariates for reference chart construction within a linear regression framework. First, a greedy forward selection algorithm was employed, changing one variable at a time until no further improvement in model fit assessed by the Akaike information criterion (AIC) is attained. This also includes a cross-validation step when comparing subsets of different covariate sizes. Second, a bootstrap-based procedure evaluating the number of significant covariates as a trade-off between model size and model fit is performed at a significance level of *p* < .05 (36).

### Construction of reference charts and curves

Considering covariates identified by the previous step of analysis, RefCurv was used for the construction of reference charts and curves based on the LMS method. The LMS method assumes that a distribution of data can be normalized by Box-Cox transformation (41). The three parameters L (*λ*, skewness of distribution), M (*μ*, median), and S (*σ*, coefficient of variation) for Box-Cox transformation were selected by choosing the subset of hyperparameters providing the lowest Bayesian information criterion (BIC) after grid search considering 5 degrees of freedom (DFs) for each hyperparameter (42) Model verification was performed by RefCurv's cross-validation facilities.

## Results

### Anthropometric data of the study patients

One hundred and twelve of 129 children (aged 0–17.92 years, mean 6.89 ± 5.32 years, median 6 years) were included in this prospective monocentric study. The distribution of sex was near-balanced with 58% (*n* = 65) male patients. Anthropometric data are presented in Table 1. Eight (8/129) patients were excluded from the analysis due to elevated liver enzymes (ALP (alkaline phosphatase), ALT (alanine transaminase), AST (aspartate aminotransferase), and/or gamma-glutamyl transferase (GGT) another nine children (9/129) due to under-/ or overweight (BMI SDS < −1.96 and >1.96, respectively).

**Table 1:** Descriptives including anthropometric data, organ size, liver perfusion, and new techniques of the study cohort (*n* = 112).

	Mean ± SD	Median	Range
Age (years)	6.89 ± 5.32	6.00	0–17.9
Height SDS	−0.10 ± 0.89	−0.11	−1.92–2.45
Weight SDS	0.13 ± 0.94	0.15	−2.04–2.43
BMI SDS	0.23 ± 0.99	0.26	−1.75–1.96
Size liver (%)^*^	109.90 ± 11.26	110.17	83.49–145.00
Size spleen (%)^*^	104.36 ± 13.09	104.00	75.36–138.96
Portal vein (cm/s)	31.03 ± 13.79	28.00	5.00–129.00
hepatic vein (cm/s)	51.80 ± 21.85	46.50	18.00–122.00
SWE (ms)	1.22 ± 0.08	1.22	1.01–1.47
SWE (kPa)	4.37 ± 0.60	4.30	2.90–6.40
SWD (m/s/kHz)	12.96 ± 1.52	12.90	9.60–18.10
ATI (dB/cm/MHz)	0.59 ± 0.07	0.59	0.06–0.81

ATI, attenuation index; BMI, body mass index; SD, standard deviation; SDS, standard deviation score; SWD, shear wave dispersion; SWE, shear wave elastography.

^*^ Liver and spleen size is given in % of normal values adjusted for body height and age.

Reasons for presentation of the study patients were unilateral kidney anomalies without impairment of kidney function (*n* = 36), urinary tract disorders without acute infection (*n* = 32), voiding disorders (*n* = 19), non-glomerular hematuria (*n* = 11), nephrolithiasis or nephrocalcinosis (*n* = 7), exclusion of precocious puberty (*n* = 3), and each (*n* = 1) with cutaneous hemangioma, dilated intestinal loops, foreign body ingestion, and with prior adrenal hyperplasia.

### Technical characteristics of ultrasound examination

The mean duration of ultrasound examination of the liver (basic ultrasound + SWE, SWD, and ATI) was 6.67 ± 2.31 min (median 6 min, range 3–21 min). In 17% (*n* = 20) of cases, the examination was tolerated with intermittent restless episodes, not prolonging the study or compromising data acquisition and quality. The remaining 83% (*n* = 92) of cases laid quietly during the examination. The mean time to last food intake amounted to 2.84 ± 1.42 h (median 3, range 1–5 h). Fifty-nine percent of patients achieved a fasting period of >2 h at the time of examination, 41% of ≤2 h. The mean abdominal wall thickness was 10.01 ± 3.11 mm (median 9.00, range 5.00 to 23.00).

### Liver size and perfusion

The size of the liver (and spleen) was normalized to age- and height-related values (%), and organ perfusion was measured in cm/s (Table 1). According to the high percentage of patients with a fasting period >2 h, the filling state of the gall bladder was moderate (*n* = 47) or high (*n* = 53) in the majority of patients (89.3%).

### Results of SWE, SWD, and ATI

Regarding SWE, ROIs were placed at a mean of 2.84 ± 0.54 cm below the skin (median 2.80, range 1.70 to 4.00 cm). Absolute numbers of measurements are given in Table 1.

### Correlation analysis

Regarding ATI, SWE (ms and kPa), and SWD, 4/112 (3.5%), 3/112 (2.7%), and 4/98 (4.1%) of measurements were winsorized, respectively. This is well below a recommended threshold of 5% (37). Moreover, the pattern of significance regarding the results of the correlation analysis did not change when using either winsorized or the original data.

Considering a power of 80% to detect a correlation of ≥0.3 regarding the analysis of ATI and SWE data, we found a significant correlation between ATI and age [r(110) = −0.52, *p* < .001], liver size [%; r(110) = 0.30, *p* = .001], abdominal wall thickness [r(110) = −0.28, *p* = .003], fasting duration [r(110) = −0.32, *p* = .001], and cooperation [r(110) = −0.34, *p* = .001] as well as between SWE (ms) and age [r(110) = 0.31, *p* < .001], liver size [%; r(110) = 0.22, *p* = .02], abdominal wall thickness [r(110) = 0.32, *p* < .001], sex [r (110) = −0.22, *p* = .02], and cooperation [r(110) = 0.28, *p* = .003—the same pattern of results was found for SWE in kPa—Table 2]. Regarding SWD, there was a significant bivariate correlation with BMI SDS [r(96) = −0.22, *p* = .03] but with no other covariate, despite sufficient power to detect a correlation of small to medium size (power 80% for r ≥ 0.33).

**Table 2:** Bivariate correlations of the new ultrasound techniques ATI, SWE, and SWD and potential influencing variables.

	ATI (dB/ cm/MHz)	SWE (ms)	SWE (kPa)	SWD (m/s/kHz)	Age (years)	Height SDS	BMI SDS	Size liver	Size liver	Abdominal wall (mm)	Sex	Fasting duration	SWD depth	Cooperation
ATI (dB/cm/MHz)	1.00													
SWE (ms)	−0.08	1.00												
SWE (kPa)	−0.07	1.00**	1.00											
SWD (m/s/kHz)	0.08	0.41**	0.40**	1.00										
Age (years)	−0.52**	0.31**	0.30**	0.00	1.00									
height SDS	0.10	−0.04	−0.03	−0.04	−0.24**	1.00								
BMI SDS	−0.15	0.10	0.10	−0.22*	0.16	0.10	1.00							
Size liver (%)	0.30**	−0.22*	−0.23*	0.03	−0.46**	0.11	0.09	1.00						
Size liver (%)	0.02	0.17	0.16	0.12	0.03	−0.01	0.29**	0.16	1.00					
Abdominal wall (mm)	−0.37**	0.17*	0.17*	−0.08	0.62**	−0.06	0.35**	0.21**	0.03	1.00				
Sex	0.03	−0.22*	−0.22*	−0.09	−0.07	−0.01	0.10	0.04	−0.18	−0.02	1.00			
Fasting duration	−0.32**	0.05	0.03	−0.19	0.42**	−0.19*	0.26**	−0.08	0.05	0.25**	0.07	1.00		
SWD depth (cm)	−0.50**	0.24*	0.22*	−0.15	0.76**	−0.16	0.38**	−0.24*	0.05	0.63**	0.01	0.43**	1.00	
Cooperation	−0.34**	0.28**	0.27**	−0.03	0.49**	−0.10	0.21*	−0.10	0.08	0.37**	−0.19*	0.12	.43**	1.00

Note: The table reports on the correlation of winsorized ATI, SWE, and SWD values with the outlined variables. Regarding abdominal wall thickness, Kendall's *τ* is reported considering all pairs of bivariate correlations, otherwise Pearson correlations are displayed. ATI, attenuation index; BMI, body mass index; SD, standard deviation; SDS, standard deviation score; SWD, shear wave dispersion; SWE, shear wave elastography. * Liver and spleen size is given in % of normal values adjusted for body height and age, SWD depth in cm.

* <.01.

** <.001.

While ATI was correlated with neither SWE (ms: r(110) = −0.08, *p* = .43; kPa: r(110) = −0.72, *p* = .45) nor SWD [r(96) = 0.08, *p* = .45], SWE was correlated with SWD (ms: r(96) = 0.41, *p* < .001; kPa: r(96) = 0.40, *p* < .001).

### Reference charts and curves

**Figure 2:**
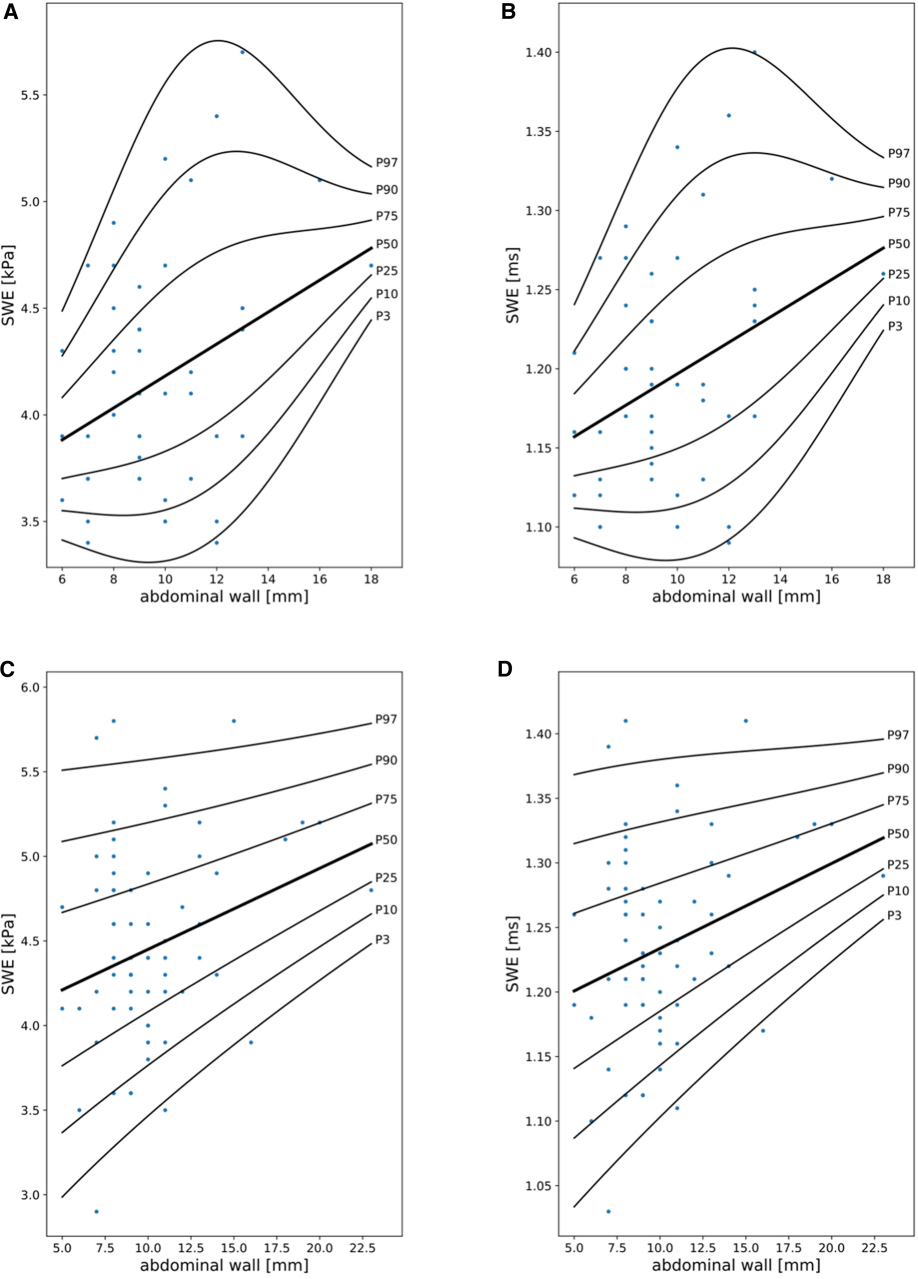
Shear wave elastography (SWE) reference curves in girls (**A,B**) and boys (**C,D**) in kPa and ms in relationship to abdominal wall thickness (mm).

SWE reference curves were fitted considering abdominal wall thickness and sex, using polynomials with 1 DF for µ and *λ* 2DFs for *σ* for both sexes (Figure 2, Supplementary Tables S6–S9).

**Figure 3:**
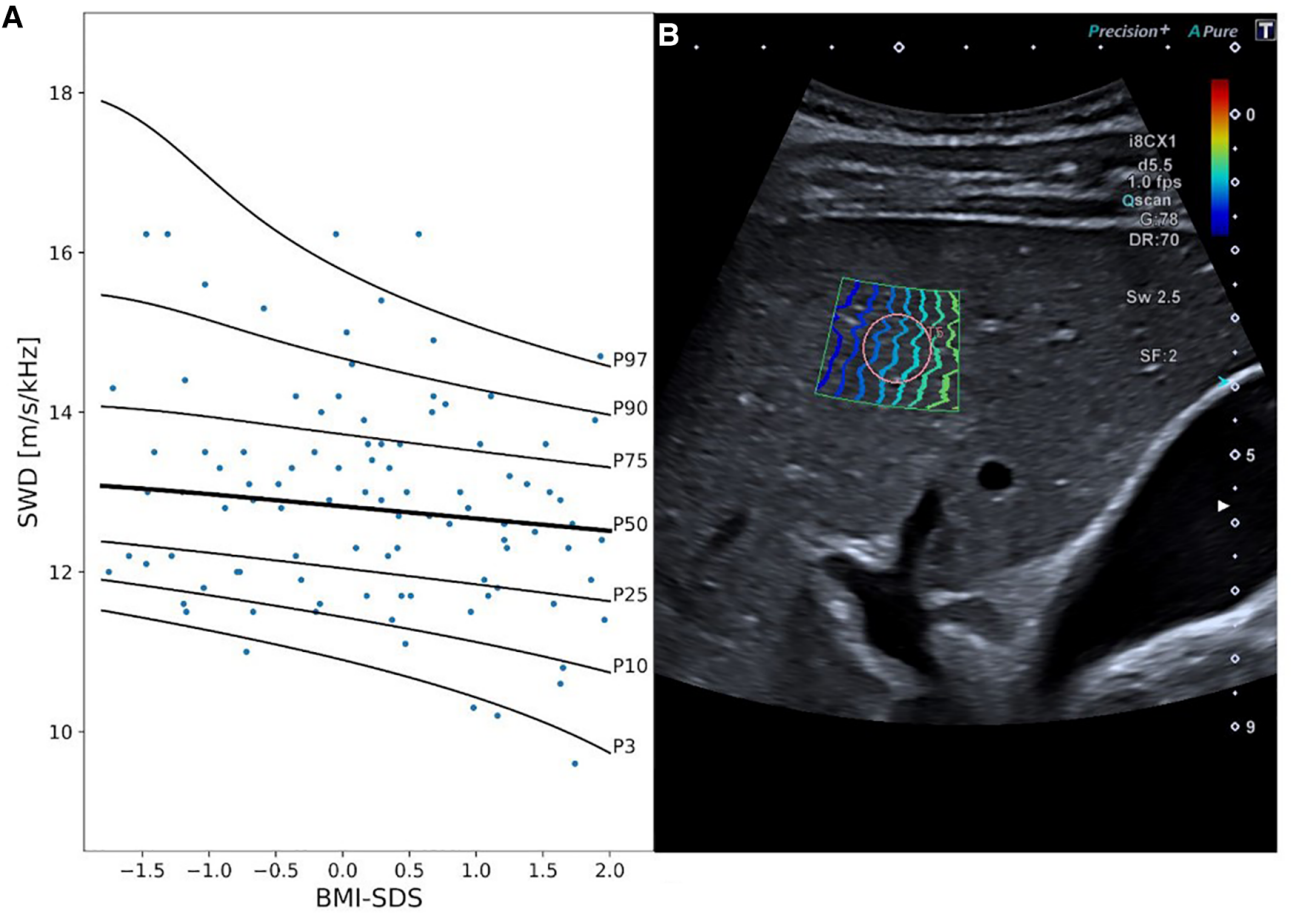
Shear wave dispersion (SWD) reference curves and exemplary 2D ultrasound SWD image. Panel A displays SWD percentile curves values in [m/s/kHz] in relationship to BMI SDS. Panel B exemplary represents an SWD measurement in a 15-year-old boy. MI, mechanical index; i8CX1, canon aplio i8CX1 transducer; d, differential tissue harmonics; fps, frames per second; Qscan, quick scan; G, gain; DR, dynamic range; Sw, shear wave frequency; SF, spatial filter.

Considering the results of the multivariable covariate selection process, SWD measurements were dependent on BMI SDS, with a decrease in SWD along with an increase in BMI SDS (best model fit considering overfitting: 0 DFs for *λ*, μ, and *σ*, Figure 3, Supplementary Table S5).

**Figure 4:**
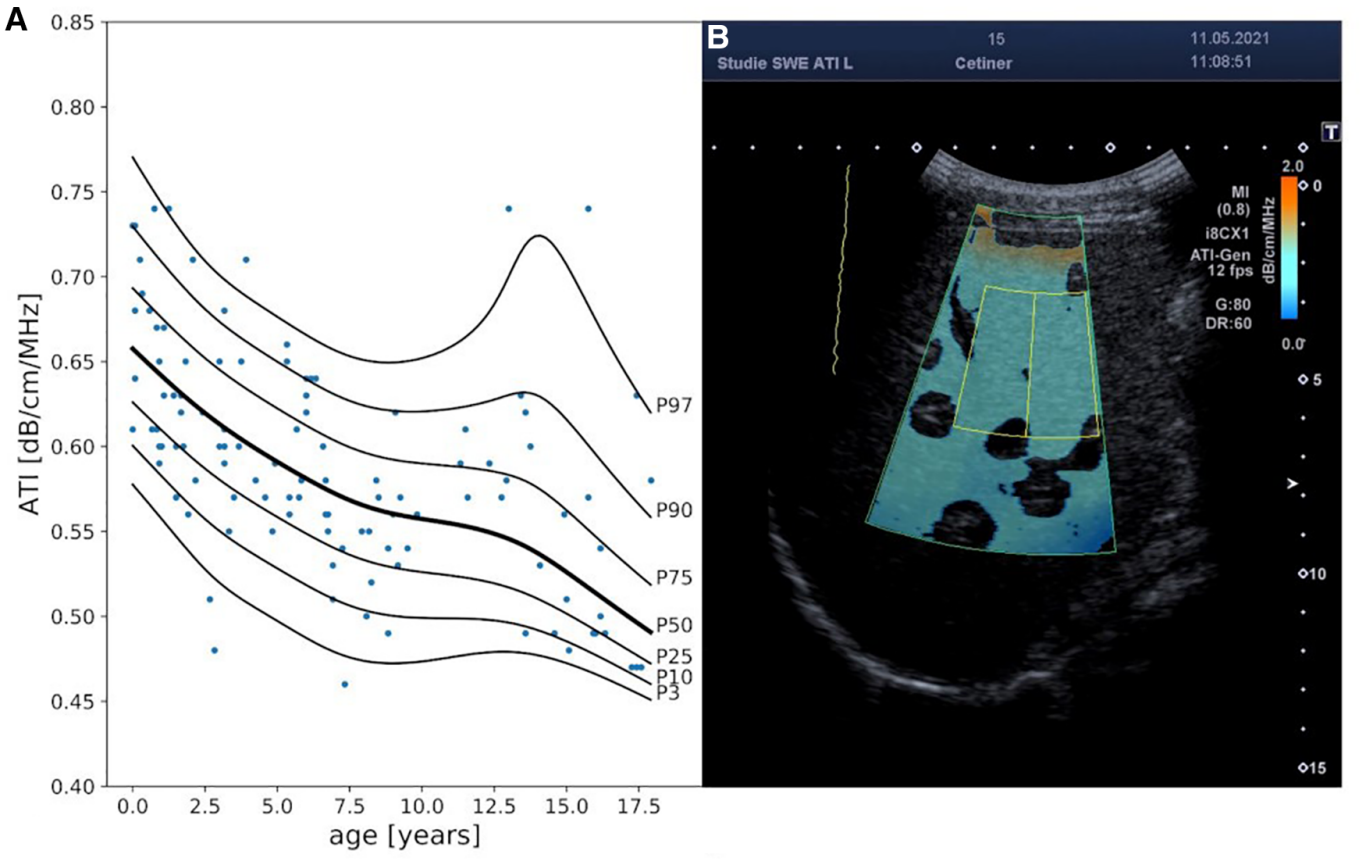
Attenuation index (ATI) reference curves and exemplary 2D ultrasound ATI image. Panel **A** displays ATI percentile curves values in [db/cm/MHz] in relationship to age (years). Panel **B** exemplary represents an ATI measurement in a 15-year-old boy. MI, mechanical index; i8CX1, canon aplio i8CX1 transducer; fps, frames per second; G, gain; DR, dynamic range.

Age was the only covariate identified by FWDselect to significantly explain the variance in ATI measurements (Figure 4, Supplementary Tables S1–S3). The best model fit (weighted against overfitting) was provided by 2DFs for the penalized splines of µ and *λ* as well as 0 DFs for the penalized splines of *σ*. As can be seen from the reference curve displayed in Figure 1, there was a decrease in ATI levels with increasing age (Supplementary Table S4).

The results from the covariate selection process were consistent when using either winsorized or the original data.

## Discussion

We aimed to establish normal values for SWE, SWD, and ATI in children and adolescents without known or suspected liver disease to improve and broaden the diagnostic options for liver evaluation by these emerging, non-invasive ultrasound tools.

### Shear wave elastography

While shear wave elastography (SWE) values were related to age, liver size (%), cooperation, sex, and abdominal wall thickness, further statistical analyses revealed that SWE values were primarily influenced by sex (higher values in boys than in girls) and abdominal wall thickness (higher values with increasing diameter). These findings support the work published in 2014 by Huang et al. (43) and in 2012 by Eiler et al. (8). A total of 509 healthy adults underwent SWE to determine liver stiffness in the study by Huang et al. (43). Whereas age and BMI had no significant effects on liver elasticity, male sex and detection depth showed a significant positive correlation. Eiler determined normal values for liver elastography (using acoustic radiation force impulse technique; ARFI) in 132 children with lower values in females without age dependency. Accordingly, we established separate percentiles for male and female patients adjusted for abdominal wall thickness. The association with sex is also supported by Galina et al. (44), but but was not confirmed by Trout et al. (16) and Bailey et al. (45). The latter studies demonstrated age-related differences in SWE, which we could confirm only by bivariate correlation, but have to negate considering multiple covariates. The absolute SWE values in our cohort (mean 4.37 ± 0.60 kPa) are comparable to already published data in children (16, 44–47) but lower than the mean values in other studies (48–50), possibly due to the usage of different ultrasound devices. Fasting duration did not significantly influence SWE in our study, as suggested by Simkin et al. (51). However, there are conflicting data from adult studies (52, 53). Anthropometric data, height-matched liver size, and liver perfusion had no significant impact on SWE.

### Shear wave dispersion

In our study, shear wave dispersion (SWD) was independent of age. Mean values [12.96 (m/s)/kHz] were slightly higher than those reported in the study of Trout et al. (16), which included 128 healthy children [mean 11.43 ± 1.75(m/s/kHz)]. However, and in contrast to SWE analysis, SWD values correlated with patients BMI SDS. Interestingly, we found a negative correlation with lower BMI scores being associated with higher dispersion values. As the prevalence of obesity is lower in children than in adults, this may explain the tendency toward lower dispersion levels in the adult population compared to children. However, data on healthy adults are limited as most studies included patients with liver pathologies (19, 20, 54). Lee et al. (55) recently published data on a small cohort including 20 healthy individuals. SWD values were comparable [12.38 (m/s/kHz)] but slightly lower than observed in our cohort.

### Attenuation imaging

Despite multiple correlations between ATI values and covariates, including age, liver size (%), fasting duration, abdominal wall thickness, and cooperation, further analyses indicate that ATI values are mainly dependent on age, with the highest coefficients in infants [mean values approximately 0.66 (dB/cm/MHz)] and a continuous decrease throughout childhood. During adolescence, we observed a broad inter-individual variation in ATI values. In late adolescence, mean values of 0.49 [dB/cm/Mhz] were observed corresponding to published data from adult cohorts (0.485–0.60 [dB/cm/Mhz] (16, 21–23, 25, 27, 30, 56);.

Cailloce et al. (15) analyzed ATI and SWE measurements in 77 children aged 0–15 years. Overall, ATI values were slightly higher [0.65 ± 0.07(dB/cm/MHz)] than those reported for adults. It was hypothesized that differences in liver architecture with a doubled layer of hepatocytes in young children compared to adolescents and adults were causative, but their data could not prove a correlation with age. However, the number of children within this young age group was limited. Another reason for the age dependency of ATI values might be the physiologically higher liver fat content in infants and pre-school children (57) and differences in liver fat content in school children in relation to prenatal weight gain and nutrition in infancy (58–60). The correlation of ATI values with age has implications for the interpretation of suspected fatty liver disease in children and should be considered before establishing and grading therapy-relevant cutoff values not only for ultrasound-based techniques but also for diagnostics with MRI (61).

The significant variance in ATI normal values in adolescence might be explained by a change in body composition during puberty and a high variation in pubertal onset (62, 63). In addition, we analyzed ATI values considering fasting duration before the examination. We could demonstrate higher attenuation coefficients in the case of shorter fasting periods prior to the examination. This is compatible with published data about daily liver fat changes (64).

### Implications for feasibility and examination conditions

Several studies discuss the feasibility of these new ultrasound techniques, especially regarding the costs of technical equipment and time-consuming examinations as well as the requirement of expert knowledge of the investigators. Carried out by only two different pediatricians experienced in ultrasound and with a standardized study protocol, the median duration of the examination (including documentation of liver size, texture, and perfusion) was 6 min and, therefore, not significantly prolonged compared to a standard liver ultrasound procedure. Examinations succeeded in all patients, with the majority (>80%) of children not showing any signs of discomfort.

We did not compel breath-hold conditions, as this is not feasible in infants and young children. Instead, the children were requested to breathe calmly. This approach is supported by data of Jung et al. (65), who did not find significant differences between examinations undertaken under breath-hold and free-breathing conditions.

The study design willfully included children with different lengths of the fasting period to analyze its influence on the study results. Moreover, a longer fasting state might have a negative impact on patients` cooperation, at least in infants, impeding the success of the examinations. Multivariable analysis negated a significant relationship between SWE, SWD and ATI results and the length of the fasting period, indicating that fasting duration is of minor importance for the analyzed parameters.

## Limitations

Limitations of our study include a limited sample size implying a lack of statistical power regarding the analyses of the importance of covariates on ATI, SWE, and SWD values. However, we had sufficient power to detect medium effect sizes. Moreover, the sample size of the present study also implies a limited coverage in terms of the range of covariate values considering the studied ultrasound parameters. This especially applies regarding SWE values in late adolescent girls (Figures 1A,B).

Even though participants were selected thoroughly and checked for conditions impairing liver parenchyma and function, some participants suffered from—albeit minor—illnesses that might influence the study results.

## Conclusion

We aimed to establish normal values (percentiles) for the new quantitative ultrasound techniques SWE, SWD, and ATI. Age, BMI, sex, and abdominal wall thickness, respectively, were demonstrated to influence at least some of these techniques and were included in the analyses. Liver size and perfusion still constitute the basis of liver diagnostics in children and they do not demonstrate an association with SWE, SWD, or ATI. The development of percentiles for the pediatric population enables the evaluation of these promising techniques regarding their potential to improve diagnostics, especially in the early stages of liver disease. Children with inborn and acquired primary and secondary liver pathologies may benefit from these emerging tools as the detection of already slight alterations is of utmost importance for early diagnosis and therapy.

## Data Availability

The original contributions presented in the study are included in the article/**Supplementary Material**, further inquiries can be directed to the corresponding author/s.
