# Supplementary material for: Validation of attenuation imaging coefficient, shear wave elastography, and dispersion as emerging tools for non-invasive evaluation of liver tissue in children
Source: Front Pediatr. 2023 Apr 17;11:1020690. doi: 10.3389/fped.2023.1020690 (PMC10150017; doi:10.3389/fped.2023.1020690)
Supplement: Supplementary file 1 [file Table1.docx]

Supplementary Material

**Attenuation imaging coefficient, shear wave elastography, and dispersion as emerging tools for non-invasive evaluation of liver tissue in children**

Metin Cetiner^1^, Felix Schiepek^1^, Ilja Finkelberg^1^, Raphael Hirtz^1*^, Anja K. Büscher^1*^

**Outline**

1. Supplementary Table 1 - Covariate Selection Process by FWDselect – ATI
2. Supplementary Table 2 - Covariate Selection Process by FWDselect – SWD
3. Supplementary Table 3 - Covariate Selection Process by FWDselect – SWE in ms/kPa

Supplementary Table 1 - Covariate Selection Process by FWDselect – ATI

|  | **covariate** | | | | | | | | | | |
| --- | --- | --- | --- | --- | --- | --- | --- | --- | --- | --- | --- |
| **q** | **age** | **sex** | **height-SDS** | **BMI-SDS** | **abdominal wall** | **liver size (%)** | **spleen size (%)** | **fasting duration** | **cooperation** | **AIC** | **p-value** |
| **1** | x |  |  |  |  |  |  |  |  | -318,31 | .85 |
| **2** | x |  |  |  | x |  |  |  |  | -320,27 |  |
| **3** | x |  |  | x | x |  |  |  |  | -324,12 |  |
| **4** | x |  |  | x | x |  |  | x |  | -323,65 |  |
| **5** | x |  |  | x | x | x |  | x |  | -323,46 |  |
| **6** | x |  |  | x | x | x |  | x | x | -322,93 |  |
| **7** | x |  | x | x | x | x |  | x | x | -321,64 |  |
| **8** | x |  | x | x | x | x | x | x | x | -320,05 |  |
| ATI FWDselect results. q = size of the considered covariate subset; x = covariate selected by the FWDselect algorithm at size q; AIC = Akaike information criterion; p-value = p-value regarding the hypothesis H1(q): the model fit improves including additional covariates. | | | | | | | | | | | |

Supplementary Table 2 - Covariate Selection Process by FWDselect – SWD

|  | **covariate** | | | | | | | | | | | | |
| --- | --- | --- | --- | --- | --- | --- | --- | --- | --- | --- | --- | --- | --- |
| **q** | **age** | **sex** | **height-SDS** | **BMI-SDS** | **abdominal wall** | **liver size (%)** | **spleen size (%)** | **fasting duration** | **cooperation** | | **SWD depth** | **AIC** | **p-value** |
| **1** |  |  |  | x |  |  |  |  |  |  | | 337,506 | .29 |
| **2** |  |  |  | x |  |  | x |  |  |  | | 335,779 | - |
| **3** |  |  |  | x |  |  | x | x |  |  | | 336,093 | - |
| **4** | x |  |  | x |  |  | x | x |  |  | | 337,021 | - |
| **5** | x |  |  | x |  |  | x | x |  | x | | 337,557 | - |
| **6** | x |  |  | x |  | x | x | x |  | x | | 338,965 | - |
| **7** | x |  |  | x |  | x | x | x | x | x | | 340,767 | - |
| **8** | x |  |  | x | x | x | x | x | x | x | | 342,563 | - |
| **9** | x | x |  | x | x | x | x | x | x | x | | 344,452 | - |
| SWD FWDselect results. q = size of the considered covariate subset; x = covariate selected by the FWDselect algorithm at size q; AIC = Akaike information criterion; p-value = p-value regarding the hypothesis H1(q): the model fit improves including additional covariates. | | | | | | | | | | | | | |

Supplementary Table 3 - Covariate Selection Process by FWDselect – SWE in ms/kPa

|  | **covariate** | | | | | | | | | | |
| --- | --- | --- | --- | --- | --- | --- | --- | --- | --- | --- | --- |
| **q** | **age** | **sex** | **height-SDS** | **BMI-SDS** | **abdominal wall** | **liver size (%)** | **spleen size (%)** | **fasting duration** | **cooperation** | **AIC** | **p-value** |
| **1** |  |  |  |  | x |  |  |  |  | -260,68 | .03 |
| **2** |  | x |  |  | x |  |  |  |  | -264,02 | .55 |
| **3** |  | x |  |  | x |  |  |  | x | -264,68 |  |
| **4** |  | x |  |  | x | x |  |  | x | -264,93 |  |
| **5** |  | x |  |  | x | x | x |  | x | -265,15 |  |
| **6** |  | x |  |  | x | x | x | x | x | -263,65 |  |
| **7** |  | x |  | x | x | x | x | x | x | -261,83 |  |
| **8** | x | x |  | x | x | x | x | x | x | -259,86 |  |
| SWE (ms) FWDselect results. q = size of the considered covariate subset; x = covariate selected by the FWDselect algorithm at size q; AIC = Akaike information criterion; p-value = p-value regarding the hypothesis H1(q): the model fit improves including additional covariates. Note, analyses in kPA had the same results. | | | | | | | | | | | |
